# Living With Endometriosis: A Reflexive Thematic Analysis Examining Women’s Experiences With the Irish Healthcare Services

**DOI:** 10.1177/10497323231214114

**Published:** 2023-11-21

**Authors:** Ayisha Lightbourne, Sarah Foley, Maria Dempsey, Mary Cronin

**Affiliations:** 1 School of Public Health, 8795 University College Cork, Cork, Ireland; 2 School of Applied Psychology, 8795 University College Cork, Cork, Ireland

**Keywords:** endometriosis, Irish healthcare services, lived experiences, endometriosis treatment, chronic pain, women’s health

## Abstract

Endometriosis is an incurable chronic condition associated with debilitating pain and subfertility, affecting 1 in 10 women. The current study aims to explore the perceptions and experiences of women with endometriosis regarding the diagnosis, support and treatment options available in Ireland. It will further determine whether additional supports or improvements are needed to care well and effectively for women with this disease in the Irish healthcare system. A qualitative study design was deemed most suitable. Twenty participants, women aged 18 and over with a diagnosis of endometriosis and experience of the Irish healthcare system, were recruited through purposeful sampling to complete semi-structured, one-to-one online interviews. Data was analysed using reflexive thematic analysis, and five themes were identified: ‘dismissive attitudes normalising severe pain’, ‘inadequate health system’, ‘the impact of delayed diagnoses’, ‘lack of education and awareness’ and ‘navigating ignorance, taboo and societal views’. Insights into the experiences and needs of women diagnosed with endometriosis in Ireland were gained, and we discuss the implications of our findings for Irish healthcare services with reference to feminist health equity and recent national action plans. We propose a series of recommendations for patient-centred care models including increased access to training and education, as well as support for longer-term chronic pain management.

## Introduction

Endometriosis is characterised as the occurrence of endometrium-like tissue outside the uterus (Zondervan et al., 2020). It is an incurable chronic condition associated with debilitating pain and subfertility, affecting an estimated 1 in 10 reproductive-age women, 176 million women worldwide (Horne et al., 2017) and approximately 155,000 women in Ireland (Endometriosis Association Ireland, 2020). Endometriosis is complex in nature and has an unidentified aetiology (Horne et al., 2017). Various symptoms are associated with the condition including chronic pelvic pain, infertility, fatigue, painful menstruation, dysuria (uncomfortable urination), dyspareunia (painful intercourse) and dyschezia (difficulty in defecating). These may significantly impact a woman’s sexual, physical and mental health, as well as her social wellbeing, working life and finances (Zondervan et al., 2020). Endometriosis’ economic impact was considered in an international study by Simoens et al. (2012), which reported a total annual cost per woman of €9579. An Australian study further reported an annual spend of $16,970 to $20,898 per woman (Armour et al., 2019). The cost to the global economy is estimated at USD 80 billion/€70 billion annually (Horne & Saunders, 2019), with most costs associated with loss of productivity and absence from work (Armour et al., 2019; Simoens et al., 2012).

The availability of high-quality services and well-informed, skilful physicians, surgeons and allied professionals are essential to the appropriate diagnosis and management of endometriosis. Negative impacts associated with health and quality of life accumulate over time when correct diagnosis and treatment are delayed (Davenport et al., 2022). A Cochrane review of the accuracy of imaging for the non-invasive diagnosis of endometriosis (Nisenblat et al., 2016) concluded that while none of the imaging modalities reviewed were accurate enough to detect overall pelvic endometriosis, transvaginal ultrasound (TVUS) and magnetic resonance imaging (MRI) are currently considered the most effective diagnostic methods (see Nisenblat et al., 2016). Multidisciplinary management of chronic pelvic pain is advised to improve quality of life of those living with endometriosis (Mechsner, 2021), involving a gynaecologist, pain specialist, physiotherapist and psychologist (Zondervan et al., 2020).

### Endometriosis Care in Ireland

Multidisciplinary healthcare has not been available to women with endometriosis in Ireland. In the Irish context, to access a gynaecologist one must be referred by a general medical practitioner (GP) who acts as a ‘gatekeeper’ to specialist medical services. Ireland has an unequal, two-tiered health service, relying on public and private healthcare provision (Heavey, 2019; Murphy et al., 2020). This two-tiered, largely tax-funded model bears similarities to other international healthcare services, including the Australian health system (Murphy et al., 2020), which was the first country to launch a co-ordinated ‘National Action Plan for Endometriosis’ in 2018 (Department of Health, Australian Government, 2018).

The Irish two-tiered model is complicated by the fact that private care is commonly delivered in public hospitals by the same medical personnel; publicly funded infrastructure is used in the delivery of private healthcare (Heavey, 2019). If a woman needs to access the public service, she is likely to wait a significant period for an appointment with a gynaecologist; for example, in 2019 the waiting time in the public health service was 2 years, while the only endometriosis centre in the country had a 3-year waiting list (McCrave, 2019). Access to private health is associated with social class and income in Ireland and raises issues pertaining to health inequity, in health service usage was examined by Fourquet et al. (2019), in relation to endometriosis care; the authors reported that medical service usage was three times lower for women relying on public sector services than those using private sector services, indicating a clear disparity linked to income.

Gender-related health inequity is also a concern, and inadequate provision of health research and healthcare for women, beyond maternal/reproductive healthcare, has been evident over time in many countries (Dusenbery, 2018; World Health Organization, 2023). Galea and Parekh called for an end to the neglect of women’s health research, concluding that “a greater focus on women’s health is an imperative that will translate into improved health for all” (2023, p. 1303). Sebring (2021) has argued that the under-development of health research and health services for women is a socially constructed phenomenon rooted in medicine as a profession and practice which was largely developed for “the wealthy, white, able-bodied, heterosexual, cissex, endosex, cisgender, male subject” (ibid, p. 1954). This long history of invalidating the experiences of ‘others’ as being “all in their head” (ibid, p. 1953) has been conceptualised as ‘medical gaslighting’ (Dusenbery, 2018; Sebring, 2021). The under-provision of women’s healthcare has been compounded in the Irish context due to two powerful, conservative influences: First, the Roman Catholic church which historically provided the majority of healthcare services and played a particular, obstructive role regarding the provision of reproductive and sexual health services (Browne, 2007) and second, resistance from some medical professionals towards the development of public healthcare in favour of maintaining the two-tier public–private system (ibid). The 2006 National Women’s Council of Ireland (NWCI, 2006) position paper on women’s health highlighted the scarcity of priority and limited approach given to women’s health in Ireland arguing that Irish health policy differed from acknowledged human rights approaches and standards. The report also noted that investment in investigations and treatment for women focused on disease prevention was low in comparison with international standards (NCWI, 2006). A study commissioned by the NCWI (2019) acknowledged that a significant portion of policies and health services are still shaped around the features and expectations of male patients, contributing to the challenges women face in accessing appropriate health services. As recently as 2018, a public inquiry into a scandal related to statutory cervical screening services identified a culture of ‘paternalism bordering on misogyny’ in that service and highlighted that ‘many of the major controversies about maltreatment of patients or denial of reproductive rights in the Irish healthcare system have involved women being damaged’ (Loughlin et al., 2018).

In Ireland, significant diagnostic delays are experienced regarding endometriosis. On average, it takes 9 years to receive a diagnosis (Endometriosis Association Ireland (EAI), 2020), 2 years longer than the 7-year average noted by Zondervan et al. (2020) in their review. Indeed, Cullen (2019) quoting a long-time endometriosis patient reflecting on the Irish situation noted that: “in 30 years of living with the condition, little has changed in how it is treated.” This context of limited provision of women’s health services has led to additional significant negative consequences for women with endometriosis. In 2021, the Irish government acknowledged this when allocating funding to develop the only endometriosis centre in the country in Dublin into a specialist centre capable of treating all types of the disease, with a particular focus on advanced and complex cases, for which there was no previous service in the country (Department of Health, 2021).

The current study focuses on access to, and quality of, health services and healthcare encounters for women with endometriosis in Ireland. Drawing on the work of the NCWI (2006, 2019), it takes a feminist and health equity approach with a primary focus on women’s lived experiences. Feminist public health is an interdisciplinary domain where meaning regarding gender constructions within health matters is identified along with gender-powered relations relating to the uneven distribution of health determinants in any population (Hammarström, 1999). An important aim of feminist public health is to minimise inequalities in health by improving health equity for ‘gender-subordinated’ groups. Our study was guided by the list of top ten research priorities for endometriosis in Ireland and the United Kingdom produced by the Endometriosis Priority Setting Partnership (EPSP) (Horne et al., 2017). It is noteworthy that while a patient representative from the Endometriosis Association of Ireland was listed on the EPSP membership, there was no clinical representative from Ireland (ibid).

### Endometriosis Patients’ Health Encounter and Health System Experiences

Previous research has examined women with endometriosis’ experiences of international healthcare services, noting challenges experienced across countries. Lukas et al. (2018) identified that 45.4% of women diagnosed with endometriosis were unsatisfied with their medical support and lacked information about the condition or treatment options. A systematic review of patients’ perspectives surrounding endometriosis care (Dancet, 2014) identified that patients value patient-centredness combined with effectiveness and safety regarding their care. The authors proposed care be organised along the following nine dimensions: access to care; coordination and integration of care; information, communication and education; physical comfort; emotional support and alleviation of fear and anxiety; involvement of significant others; continuity and transition; endometriosis healthcare staff; and technical skills (p. 75–77).

International research has also examined the impact and the prevalence of negative healthcare interactions when presenting with endometriosis symptoms. Pettersson and Bertero’s (2020) meta-synthesis of 14 qualitative studies on healthcare encounters from Sweden, the United Kingdom, Italy, South Africa, Australia and New Zealand presented three fusions: “Insufficient Knowledge, Trivialising – just a women’s issue, and Competence promotes” health (p. 529). Findings from this meta-synthesis note the perceived disinterest in women’s pain reported, international variation in treatment options, and long-term impact of these on fertility options and quality of life. The meta-synthesis also noted the positive experiences reported when women were transferred quickly to specialist care (ibid). Fernley’s (2021) analysis of online public accounts in Australia of experiences leading to endometriosis diagnosis produced some themes similar to Pettersson and Berterös (2020) meta-synthesis; it also reported how a significant majority of women remained unaware of endometriosis for some time. Rowe et al. (2021) further recommends longer-term care planning and a collaborative approach to care incorporating women’s self-knowledge and expertise. Evans et al. (2021) echoed many of the above points, adding that women with endometriosis felt “Let down” by doctors as a result of experiencing medical gaslighting and needed to fight to be heard (p. 2099).

Sbaffi and King (2020) published the only study on endometriosis to date that includes Irish women. In a mixed-methods survey, they explored the role of the internet in supporting endometriosis diagnosis and treatment. Findings reported that comprehensive, reliable treatment and diagnosis information from health practitioners remains inadequate, and as a result, patients are turning to ‘online peer-to-peer information exchange’ where endometriosis is not minimised, dismissed or disregarded completely. The authors found the information which endometriosis organisations provide online is trusted by women, and the peer exchange process provides acknowledgement and validation which is scarce in other areas of patients’ lives.

A recently published comparative study in which patients across 13 European countries benchmarked their endometriosis care (Dancet et al., 2023) included Ireland; however, there was insufficient Irish data for comparison highlighting the need for research on the Irish context. This research aims to examine the experiences of 20 women with endometriosis in the Irish healthcare system in relation to diagnosis, treatment and support and to amplify their recommendations for service improvements. This work is timely as a National Endometriosis Framework (NEF) is under development (Department of Health, 2023), and a new endometriosis service will include two nationwide referral centres along with four specialist hubs (ibid). The NEF and its associated services were not operational at the time of data collection.

## Methodology

### Study Design

This qualitative study used individual, online semi-structured interviews to collect data. As the focus of this study was on the experience of living with endometriosis, the research team undertook an inductive reflexive thematic analysis informed by Braun and Clarke (2006, 2019, 2022). Analysis centred on latent meaning in the data. The research team approached the data with a constructionist perspective; that is, “meaning and experience was interpreted to be socially produced and reproduced via an interplay of subjective and intersubjective construction” (Byrne, 2022, pp. 1395–1396).

### Participants Recruitment, Procedure and Data Collection

Through purposive sampling, women who met the study inclusion criteria of being aged 18 years and older, having a self-reported medically confirmed diagnosis of endometriosis via laparoscopic surgery and having experience of the healthcare services in Ireland were invited to participate in the study. Of the 25 women who expressed interest, 20 met the inclusion criteria. Participants ranged in age from late teens (18+) to late fifties and most were based in urban locations. Time taken to establish a diagnosis ranged from 2 to 32 years, with an average of 6 to 7 years. Most participants had experience of both the public and private Irish health services, with the majority choosing the latter at consultation level, after initial presentation at GP or Emergency Care.

After institutional ethical approval was granted, the Endometriosis Association of Ireland (EAI) aided study recruitment by emailing study information to their members. People who were interested in hearing more about the research contacted the first author directly. If they fulfilled the inclusion criteria and gave consent to participate, they were invited to take part in an individual online recorded interview, via Microsoft Teams. Prior to the interview, each participant was sent an information sheet detailing the purpose of the study and a consent form to be signed and returned to the first author via email; verbal consent was additionally obtained at the beginning of each interview. Following settling in questions, the researcher adopted a fluid approach to interviewing and asked each participant if they would “tell me about your experience of endometriosis.” This allowed participants to choose their own starting point to share their story. Priority was given in interviews to following participants’ narratives though at times the researcher drew from a brief interview guide which comprised questions such as “What was your experience of being diagnosed?” At the end of the interview, participants had further opportunity to provide any additional information they wished and ask the researcher questions about the study. The interviews ranged between 30 and 60 minutes in duration and were conducted in the summer of 2021.

### Data Analysis

All interviews were read several times and transcribed verbatim with initial notes being logged by the analysts who were informed by public health or psychological perspectives and all of whom had completed prior research into women’s health issues. Each transcript was anonymised, and each participant was given a pseudonym. While the first author (AL) completed most of the analysis, to enhance quality measures and interpretative depth, all authors were involved in transcription, coding and discussions of themes. Data analysis followed an iterative, collaborative process with a dual focus of immersion and distancing which allowed time for reflection on codes and themes (Braun & Clarke, 2022). For example, after two interviews had been coded line by line, three members of the team met to discuss the coding process. They met again after the focus coding process was completed on seven transcripts to reflect on codes within, and across, transcripts. Through ongoing team discussions, synergy between codes was noted and codes were collated to develop themes. At this point in the analytical process, three members of the research team (SF, MD and MC) each reviewed three transcripts independently to consider patterns of meaning across data associated with themes. As some of the data straddled several themes, refining theme names took time and multiple collaborative discussions. Bracketing (Tufford & Newman, 2012), through use of reflexive memos and discussion, was used to process assumptions brought to, and insights about, the data. All team members refined, and agreed to, the final five themes.

## Findings

In this article, we focus on findings related to healthcare encounters and health service experiences. We identified themes pertinent to examining the Irish context and detail these experiences within the following five themes: ‘dismissive attitudes normalising severe pain’, ‘inadequate health services’, ‘the impact of delayed diagnosis’, ‘lack of education and awareness’ and ‘navigating ignorance, taboo and societal views’.

### Dismissive Attitudes Normalising Severe Pain

Dismissive attitudes were identified through the participants’ narratives of their interactions with healthcare professionals. This included not feeling listened to, or taken seriously, when communicating their concerns about their symptoms. Feeling of being dismissed was experienced at both the pre-diagnosis and post-diagnosis stages. For example, Amy recalled a response from her GP after she shared her concerns in an appointment:

Oh, you’re just stressed, you need to relax …. you know sometimes you just need to embrace feeling unwell.

This advice to ‘embrace feeling unwell’ is an extreme example of the inappropriate dismissal of pain by a healthcare professional, possibly implying that symptoms have been brought on, at least in part, due to the stress being experienced by Amy. Unfortunately, this normalisation of severe pain permeated our data. Participants described how their painful symptomatology was not acknowledged and debilitating menstruation was normalised in healthcare encounters. Below, Karen outlines advice she received from her GP:

I went to my GP, and they said, “bad periods are normal, pain’s normal you know, and you should be bleeding when you have your period.”

This pain normalisation undermined Karen’s reality, as she has reported heavy bleeding and severe pain during her periods. In addition to early dismissal at GP level, this perceived dismissive attitude continued during treatment for Lila. She detailed her frustration at the minimisation of her endometriosis by a consultant, post examination:

The consultant … was like “look there’s endometrium there, nothing major, nothing to be overly worried about, plenty of lesions and endo on the right-hand side.” I remember …. just being, you know f** this, how bad does it have to be for it to be something?

Feeling dismissed by healthcare professionals became a barrier for many participants to seek further treatment. Ongoing challenges in accessing appropriate support and treatment are presented in the next theme.

### Inadequate Health Services

Participants believed that the health services in Ireland were failing women with endometriosis. Examples in the data spoke to experiences at all healthcare levels, including GPs visits, consultation, surgery and aftercare. Participants described a consistent lack of support and information regarding symptom management and treatment. The disparities between the public and private systems were also noted, and the public healthcare system was deemed unsuitable when seeking appropriate care. Even with access to private care, Karen described seriously considering seeking treatment abroad:

At one stage I was looking at going abroad – I was looking everywhere because I was like this is like this is bad (emphasis) like.

While some women we interviewed had initially engaged with public healthcare provision, they quickly sought out alternative options, including travelling abroad. Karen considers herself to be ‘extremely lucky’ to now have private healthcare, a sentiment that was shared by others, and highlights the privilege of having far earlier access to consultant-level care.

Susie was able to obtain free scans while living abroad, which she shared with her GP upon returning to Ireland:

I went to see my GP and he was amazed, saying “this is incredible detail, and the quality of the scans … you wouldn’t have gotten that here”, he said.

Here, the GP further confirms the sentiment shared by the women we interviewed that high-quality care accessible elsewhere is yet to be available in Ireland. Adding to this, the lack of a dedicated endometriosis centre in Ireland meant participants had to retell their story many times to various medical practitioners. This was viewed as inadequate, frustrating and, at times, upsetting. For example, Maura, who was told her only option would be a hysterectomy at the age of 26, considers it the ‘luck of the draw’ what kind of advice is given:

They said to me “we can’t operate on you again like the next operation will be a hysterectomy,” you know. So that … that is kind of an awful thing to face at 26/27, you know you’re kinda like oh god … it is a life changing thing to happen you know. So I do think that there’s kind of, it is the luck of the draw with what doctor you see and how they feel about it.

The absence of expert surgeons was troubling to participants, as was the nature of treatments being offered, specifically the continuing use of ablation techniques. Grace expressed frustration that excision surgery was not yet commonly offered in Ireland:

I mean obviously, if there’s a well-established gold standard (excision surgery) … like why isn’t here and why isn’t that our standard ...?

This frustration at a lack of expert surgeons in Ireland was one example of how women felt let down by the healthcare system. Amy shares how she internalised the inadequate care she received, the impact of which was her questioning herself:

I feel let down. They’re days I feel let down by my GP, by the medical system, by resources that are available, let down by myself, that like I said I didn’t just Google a few symptoms and have that pop up. Because you just get convinced that “no I’ve checked it all out and everybody says I’m OK so I must be OK.”

Judy further demonstrates how women with endometriosis navigate inadequate services and try to avoid delays:

I would never miss an appointment. Even if you were having two or three months where you were kind of stable, you’d be afraid to miss your appointment because things can change so quickly and then your next appointment could be a year later.

This account illustrates an additional burden for some women who feel obliged to take whatever service is provided, regardless of whether it meets their needs at a point in time. This theme demonstrates the additional steps required to seek out appropriate care in the Irish healthcare services, the inflexible services that are offered and the variation in advice and treatment provided.

### The Impacts of Delayed Diagnosis

The third theme identified the experience of significant delays in receiving a diagnosis after presenting with symptoms and the impacts thereof on quality of life, personal finances and reproductive health. Lila recounts the years it took from first symptoms to formal diagnosis:

It was about 14 to 20 (years of age). The guts of seven years and that’s just wrong y’know. Why should I have to suffer for seven years with no acknowledgement?

Here, Lila notes the suffering that ensued while awaiting a diagnosis, as well as the dissatisfaction in the delay. This further highlights the personal and psychological impact of the inadequate healthcare services outlined in the previous theme.

Paula, who had been initially diagnosed in her home country in Europe, described seeking follow-up care in Ireland:

I don’t have private insurance, so it actually took me one/one and a half years to get an appointment with the gynaecologist in Ireland. In the meantime, from my own expenses, I was paying to go to my [home country] gynaecologist.

This quote also points to financial costs borne by the participants in an attempt to manage their condition. Having to travel abroad for treatment due to delays in the Irish service further highlights the burden of self-management for the condition.

A smaller cohort of participants detailed the challenges of misdiagnosis with conditions with similar symptoms:

They thought originally that I might have had Crohn’s, so I’ve had 3 colonoscopies and 2 endoscopies. I’ve had the barium swallow test, a pillcam test, MRIs, ultrasounds. I’ve just had so many tests done. (Ola)

This account exemplifies inconsistencies in considering a diagnosis of endometriosis, resulting in a delay in appropriate treatment, as well as the considerable burden of undergoing so many investigations.

In addition to disruption arising from attending appointments, both Cora and Amy reflected on the impact of the delayed diagnosis on their mental health:

Looking back, I can see how much I suffered with anxiety and depression, and I know that's linked in with endometriosis and it was … I always felt there was something wrong with me but couldn’t put my finger on it … (Cora)

I think to just have a name to how crap I felt … I was like it was not all in my head I knew it, like I told you all and I knew it … (Amy)

These quotations highlight the psychological impact of delayed diagnosis and the subsequent relief and sense-making that can take place once a diagnosis has been established. Finally, many participants articulated their concerns and difficulties regarding fertility and felt further let down by the healthcare professionals as a result. Quinn’s quotation below provides an emotive example of the impact of delayed diagnosis, specifically on her fertility and her future:

It’s just for me it’s just it’s extremely upsetting because like the odds of me ever having a child now are like really extremely low.

This theme details the financial and personal impact of securing a formal diagnosis. In addition, this theme illustrates the concerns raised by participants as to future physical and mental health consequences of a delayed diagnosis.

### Lack of Education and Awareness

The lack of education and awareness in Ireland surrounding endometriosis and endometriosis care unfolded through the participants’ narratives in relation to both healthcare professionals and women themselves. The need for more education and awareness for medical professionals to improve diagnosis, support and treatment of patients with endometriosis was prevalent. Riley further described her frustration at the lack of inquiry from GPs:

No doctor ever really scratched the surface, and they never really asked any questions, and maybe I didn’t ask questions and all that kind of thing but no GP ever kind of said you know you … like you’re a classic case.

Here, Riley questions where the responsibility lies between herself and her GP, to ask the probing questions regarding her condition. In contrast, Delilah placed clear responsibility on medical professionals being educated to recognise classic endometriosis symptoms and thus ensure earlier detection in primary care:

Period pains shouldn’t be debilitating, and they should understand that that’s not normal ...

Other participants focused on the necessity to educate themselves about endometriosis and believed they had not received support or information from medical professionals on how to best manage symptoms and pain. For example, some noted learning from their own research or peers about drug management or complementary therapies which could have empowered them to be better able to manage and live with their condition, had they heard this from their doctors. Judy reflected on what she perceived as ‘naivety’ at the beginning of seeking treatment:

At the time you go listen to what your consultant’s telling you. I think it takes a long time to advocate for yourself. I’d be much better now but that’s with age and like it's kind of too late now.

Judy conveys her sense of personal development linked with navigating the medical system but also implies she feels it is too late to undo damage caused earlier in her treatment. Many participants spoke explicitly about the importance of self-advocacy. For example, Ivy is adamant her own daughter will not have the same experience but also articulates a sense of persistent limitations within the Irish health services:

You need to advocate for yourself, especially if you’re a woman when it comes to your health. That’s the positive if anything. I have a daughter …. I could say there’s no way she’ll go through what I went through but then you know there’s no solution in Ireland either.

In some cases, participants attributed securing their first referral to a consultant to expressing their emotional state overtly to professionals:

I think the only reason why I even got referred to the [Private Hospital] was because I literally was just in floods of tears in her office … just like I can’t go on like this you know. Grace

This theme has emphasised the need for education and awareness around endometriosis. The element of self-education by women is particularly interesting as it highlights how within the Irish context the patient is obliged to be very proactive in educating and advocating for themselves to ensure appropriate care and treatment for endometriosis. This requires a level of education, confidence, skills and resources which may not be available to all, with consequent implications for health equity.

### Navigating Ignorance, Taboo and Societal Views

As a result of a lack of specialist services and understandings noted above, the final theme details the wider impact of this lack of awareness and understanding of endometriosis. Participants identified the impact this had on their relationships with family, friends and colleagues, who were unable to understand and therefore support their efforts to seek appropriate care. Riley acknowledges her own lack of education on the illness until faced with the condition:

I never knew about endometriosis … I just feel sometimes quite embarrassed because I’m … an educated person in the world and just think “how did I not know this?”

While some believed this was their own responsibility, others attributed it to the historical taboo in Ireland related to reproductive and sexual health:

I suppose in Ireland you don’t really talk about these things either, so I sometimes felt you know there was nobody really close to me that I felt kind of understood. (Ellie)

Beyond taboo, there was a sense that the lack of specialist care and diagnosis opportunities meant pain was normalised by family and peers, and therefore diminished. There was frustration that endometriosis pain was equated to regular experiences of periods and therefore deemed tolerable:

People just seem to think that they know what that is for every woman, and I think they sometimes think “well I get my period and I’m grand so can you just not handle the pain” as if it’s the exact same which it’s obviously not you know. (Grace)

In response, participants called for more input in schools to educate everyone on endometriosis and, especially, to ensure young girls are aware of the symptoms. Some felt that education alone was not enough and that it should be accompanied by an increase in resources and treatment, including screening.

In addition to social and relational impacts, many participants described the impact living with endometriosis had on their experiences of work. Access to healthcare appeared to be mediated through work experiences, which resulted in issues around sense of self and a further lack of support, a recurring issue related to employment options. For example, Riley spoke about her attempts to overcompensate in work to avoid being viewed as ‘that sick person’, indicating her perception of negative societal views of ill people:

With work it definitely affects it in terms of if I’m missing two days of the month, having to work extra, trying to cover, trying to not be that sick person, trying to not be you know the person cancelling on things.

Fran also suggested negative views of absences from work when sick:

I had to take extended sick leave from a job I only started, I mean that looks absolutely terrible, then you feel like you can’t progress maybe the way that you want.

This concern regarding career impact was shared by a number of participants who said their careers had not progressed as they would have liked due to having to cut down on their workload. Another participant described keeping her diagnosis of endometriosis to herself, suggesting there was little benefit in disclosing it to her male managers, particularly given the turnover of people in her company. This reflects a further gendered element within the Irish context and disregard for women’s health, whereby women do not feel like they can openly talk about their diagnosis, as it is still seen as a topic which is not appropriate to discuss. Many women opted for self-employment to mitigate the impact of accessing healthcare.

## Discussion

To our knowledge, this is the first qualitative study of its kind to solely examine the experience of accessing healthcare and treatment for endometriosis in Ireland. This study contributes to the global calls for more timely diagnosis (Davenport et al., 2022) and wider access to specialised treatment (ESHRE, 2022), as well as highlighting the impact of ineffective education and awareness on quality of life (Fernley, 2021), and advances nuanced awareness of endometriosis within an Irish context.

It is evident from international research that the healthcare experiences of women with endometriosis, across many countries, are remarkably similar, supporting Sebring’s argument of a normative, male bias in medical practices (Sebring, 2021). Our findings further detail the sub-standard experience of healthcare interactions and encounters pertaining to endometriosis care and provide insights into the personal consequences of sub-standard development of women’s health services in Ireland. The Irish experiences detailed in our findings add weight to previous literature regarding dissatisfaction with support offered by healthcare professionals (Lukas et al., 2018) and variation in treatments offered (Pettersson and Berterö, 2020). Further, the commonly reported feeling of being ‘let down’ by healthcare services in Ireland echoed experiences of women in the Australian context (Evans et al., 2021). These findings also support those of Dancet et al. (2014) which reported difficulties in accessing care, the need for improved information and education surrounding diagnosis and treatment, and more medical training and expert surgeons.

### The Irish Two-Tiered Health System: Implications for Endometriosis Care

While our findings provide further voice to the experiences of women globally, it also elevates recognition of the issues within the Irish context. The majority of participants in this study had accessed private healthcare and a feeling of gratitude was common, with many describing themselves as feeling ‘privileged’ and ‘lucky’ to be able to do so. This raises significant questions about the morality of the Irish two-tiered system, as previously discussed by Heavey (2019). Having accessed private care, many, but not all, felt satisfied with the care they received; however, concern around expert surgeons and the availability of excision surgery was an issue for women whether they had used the private or the public service. In comparison to international standards regarding diagnosis and treatment from similar healthcare services in Europe (ESHRE, 2022) and Australia (NAPE), participants reported varied treatment history and advice they had received, implying a lack of established procedure in healthcare services. Notable examples include advice from a GP to ‘embrace feeling unwell’ and the suggestion to a woman in her mid-twenties that a hysterectomy was the only remaining treatment option. These responses raise questions about appropriate communication and patient engagement. Travelling abroad for treatment was noted throughout the interviews. While ‘medical tourism’ has been reported previously in relation to women’s health in Ireland (Gilmartin & White, 2011), our findings confirm this phenomenon extends to endometriosis care. This may have further economic implications not previously noted in international research (Armour et al., 2019).

The need for significant financial means, usually in the form of private health insurance, to avoid long waiting times to access endometriosis care at consultant level, raises a number of issues for healthcare equity and feminist public health. The literature on feminist public health (Hammarström, 1999; Rogers, 2006) provides an opportunity to further examine health inequities within the Irish healthcare system and contribute to policy surrounding women’s health. To ameliorate the historical under-funding of patient-centred women’s healthcare and the conservative healthcare culture, the Irish state should consider the impact of these various factors relating to women’s health inequity as intersecting barriers, which require a substantial response; this point is applicable also to other countries with similar conservative histories. Given the historical treatment of women and reproductive and sexual health in the Irish context (Loughlin et al., 2018), the continuing under-provision of women-specific health services including for the treatment of endometriosis further contributes to the mistreatment of women in Ireland. All healthcare providers, public and private, have the opportunity to address this by ensuring that care models based on evidence of best practice are adopted, and the development of a National Endometriosis Framework (NEF) provides a vital opportunity for the design and establishment of an evidence-based service, along with more comprehensive, women’s health–oriented education for healthcare professionals in Ireland.

### Education, Treatment and Resources: Championing Best Practice

The variation in treatment, advice given and understanding reported by the women in our study raises questions regarding responsibility, care planning and education in the Irish context. Participants expressed a burden of self-care, self-education and advocacy to secure appropriate medical treatment and complementary care and strongly recommended that more education and training for healthcare professionals was needed. As noted in our literature review, Pettersson and Bertero’s (2020) meta-synthesis highlights the importance of interactions with knowledgeable, trusted healthcare professionals as a protective factor in management of endometriosis. Recent progress by the Department of Health (2022) on specialised care planning is welcomed as a starting point in ensuring standard procedures and care planning are implemented nationally. International action plans offer key insights into the coordination required to implement a successful care plan. For example, the Australian National Action Plan on Endometriosis (NAPE) (2018) has evidenced the positive impact of increasing awareness and education and improved clinical care, and an investment in research funding has resulted in marked improvement in access to care across the country. This example of good practice, in which patients are at the centre of policy and service development, presents important global examples of good practice for health services yet to establish a national action plan, including Ireland. The findings of our study contribute to the evidence basis required to successfully implement the national endometriosis framework (NEF) for Ireland and respond to recent calls by advocacy groups to centre women’s voices in the implementation of the NEF in Ireland (Griffin, 2023).

While the experience of healthcare professionals was not included in the current study, international research with healthcare professionals outlines self-reported limitations in expertise and a need for further training (Rowe et al., 2021; Young et al., 2015; Young et al., 2017). Further training and resources for healthcare professionals in Ireland could improve speed of diagnosis and treatment for young girls and women presenting with symptoms, thus alleviating the negative consequences of diagnostic delay. Beyond diagnosis and acute medical procedures, person-centred, evidence-informed care planning is required to ensure women are supported in the management of endometriosis in Ireland. Our participants detailed changing care needs they have required while living with endometriosis, including but not limited to pain management, fertility support, surgery and psycho-social support. Recent work by Agarwal et al. (2019) recommends the adoption of a long-term, patient-focused and multidisciplinary chronic care model, including complementary therapies such as nutrition and physical therapy, to improve clinical outcomes and quality of life (Agarwal et al., 2019). Additionally, participants identified a distinct lack of education, awareness and information provided on receiving a diagnosis, which resulted in feelings of frustration and regret. This extends existing research on awareness of endometriosis to the particular socio-political Irish context. Menstrual education in schools should integrate education on endometriosis into the curriculum, to ensure girls going through puberty are aware of normal and abnormal reproductive cycles. This has been evidenced in research conducted in New Zealand (Bush et al., 2017) and Australia (Armour et al., 2022) which reported that menstrual education interventions increase awareness of endometriosis and early presentation at healthcare services. Further, recent legislation in Spain covering menstrual leave for women experiencing pain explicitly acknowledges the impact of endometriosis on quality of life and productivity loss (Bello, 2023). This is a positive move towards gender equity in the workplace and provides an example of opportunity for wider awareness and support outside of the healthcare services for people with endometriosis, particularly considering the economic consequences noted by Armour et al. (2022) and detailed in our findings.

### Study Strengths and Limitations

This study is the first to present insights regarding women’s experiences of accessing healthcare and navigating health services for treatment of endometriosis in Ireland and provides a patient-centred evidence basis for policy and practice concerning quality care for endometriosis. A limitation of the current study is that participants had more experiences with private, rather than public, healthcare. Participants were also members of the Endometriosis Association of Ireland, which indicated access to existing resources and support offered by this group. Furthermore, all but one were Irish nationals, prompting questions as to whether it may be even more challenging for non-Irish nationals to navigate the complex Irish health system and would they have the means to research, self-educate and self-advocate as was necessary for our participants. For future research, it would be important to recruit participants who are representative of both public and private service users, so all women accessing the Irish healthcare system for endometriosis care are represented.

## Conclusion

The findings of this qualitative research present insights into the impact of navigating the two-tier Irish healthcare service and the consequences of under-provided and under-funded healthcare for women living with endometriosis. There are many challenges evident within these services including a lack of awareness of patients’ symptomatology in both medical and cultural contexts resulting in the normalisation of women’s pain, delayed waiting times for diagnosis and restricted access to best practice in medical treatment and holistic care. We make recommendations for patient-centred services that provide up-to-date, evidence-based practice including diagnostic and treatment interventions and effective management of chronic pain, along with resources to educate patients, healthcare providers and the public, to improve the lived experience and care of women with endometriosis in Ireland and other countries, especially those in which conservative influences have undermined the provision of quality reproductive and sexual health services for women.
